# Research Progress on Contrast-Enhanced Ultrasound (CEUS) Assisted Diagnosis and Treatment in Liver-Related Diseases

**DOI:** 10.7150/ijms.101789

**Published:** 2025-02-10

**Authors:** Xiaoqing Ying, Shangxin Dong, Yuanyuan Zhao, Zhishui Chen, Jipin Jiang, Huibo Shi

**Affiliations:** Institute of Organ Transplantation, Tongji Hospital, Tongji Medical College, Huazhong University of Science and Technology; Key Laboratory of Organ Transplantation, Ministry of Education, Chinese Academy of Medical Sciences; NHC Key Laboratory of Organ Transplantation, China.

**Keywords:** contrast-enhanced ultrasound (CEUS), liver-related diseases, liver transplantation, evaluation, clinical practice

## Abstract

Liver-related diseases, such as hepatocellular carcinoma (HCC) and cirrhosis, are globally prevalent and significantly contribute to mortality rates. Despite the availability of various imaging techniques for liver evaluation, a consensus regarding the selection of an accurate and safe method remains elusive. As a non-invasive imaging approach, the effectiveness of contrast-enhanced ultrasound (CEUS) in assisting the diagnosis and treatment of liver-related diseases has been established. Compared to conventional methods, CEUS offers notable advantages, including high safety, convenience, accuracy, and cost-effectiveness. Recent advancements have demonstrated the expanded utility of CEUS in liver-related diseases. In addition to diagnosing focal liver lesions, CEUS is increasingly employed for guiding local treatments, assessing liver transplantation suitability, and planning surgical interventions. However, its application requires caution due to the high technical proficiency demanded of operators, time-sensitive imaging processes, and susceptibility to visual interference. This review summarizes the current applications and recent advancements in CEUS-assisted diagnosis and treatment of liver-related diseases, explores its future potential, and proposes possible improvements. The objective is to enhance the accuracy and versatility of non-invasive liver assessments and provide a reference for the broader and more effective utilization of CEUS in liver disease diagnosis and management.

## 1. Introduction

**Figure 1:**
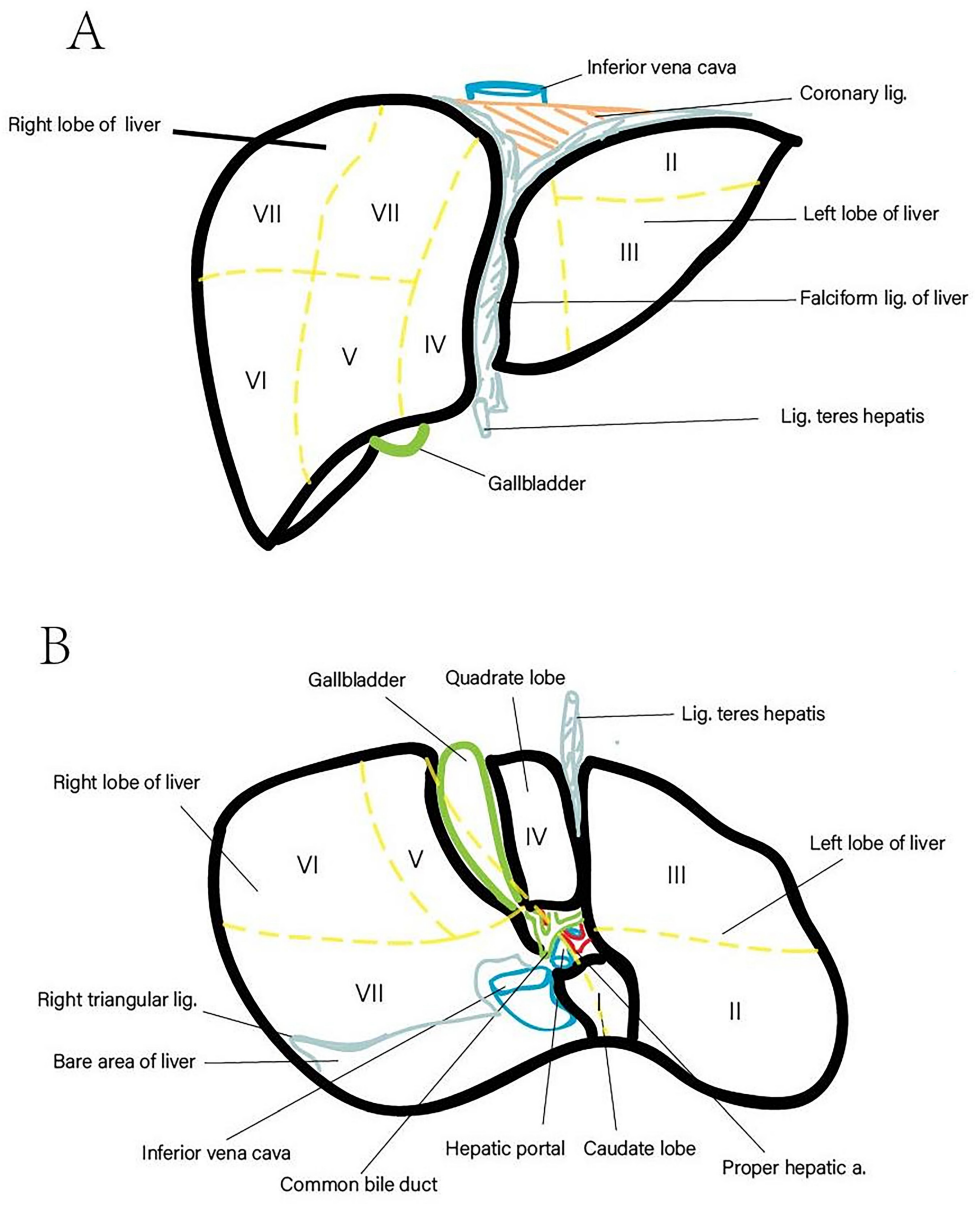
** Liver Anatomy (Lobed and Segmented).** (A) Diaphragmatic surface of the liver. (B) Visceral surface of the liver.

The liver, the largest gland in the human body, constitutes approximately 2.5% of total body weight and is responsible for various critical physiological functions, including nutrient metabolism, immune system support, blood volume regulation, and xenobiotic compound decomposition 1, 2. Anatomically, it is divided into five lobes and eight segments based on the distribution of the portal vein and hepatic vein (Figure 1). Recent statistics indicate that liver-related diseases result in approximately 2 million deaths annually, accounting for 4% of total global mortality. The primary causes of these fatalities include complications associated with liver cirrhosis, viral hepatitis, and hepatocellular carcinoma (HCC) 3. Notably, HCC is characterized by a high case fatality rate, with GLOBOCAN estimating 905,677 new cases and 830,180 deaths related to HCC in 2020. Cirrhosis is present in 80-90% of HCC cases, irrespective of etiology, underscoring its role as a significant risk factor for HCC 3. Liver-related diseases also impose substantial economic burdens on patients. Whether treated through surgery, pharmacotherapy, or monitored with routine examinations, the associated costs are considerable 4. For instance, liver-related expenditures in the United States amounted to $32.5 billion in 2016, with inpatient and emergency department care comprising two-thirds of these expenses. Over the past two decades, healthcare spending has increased by an average of 4% annually, primarily due to hospital-based services 3, 5. Early diagnosis and treatment are critical to managing liver-related diseases effectively before irreversible liver damage occurs. Hence, employing accurate diagnostic and assessment methods is imperative, as such approaches can substantially reduce both mortality rates and economic burdens.

Routine methods for liver examination include laboratory tests, imaging modalities, and tissue biopsy pathology. Compared to other methods, imaging techniques offer several advantages. First, they are non-invasive, posing no physical harm to the liver. Second, imaging provides rapid results, enabling timely diagnosis and treatment 6. Third, these methods enable morphological assessments, which can detect abnormal changes as small as two centimeters with a positive detection rate of up to 80%. Consequently, imaging is a highly sensitive and early detection modality capable of evaluating liver fibrosis and identifying focal lesions.

Currently, common imaging modalities for liver evaluation include ultrasound, computed tomography (CT), magnetic resonance imaging (MRI), and contrast-enhanced ultrasound (CEUS). Among these, CEUS is a novel, non-invasive technique that enhances blood flow signal detection through the intravenous administration of ultrasound contrast agents. This technique significantly improves the specificity, sensitivity, and resolution of ultrasound imaging, facilitating more accurate clinical assessments. CEUS employs microbubble contrast agents, which remain within the vascular system, thereby enabling safe and precise evaluation of focal liver lesions 7, 8. Additionally, CEUS can assess the enhancement and regression patterns of tumors, providing critical insights into their growth characteristics and nature. Unlike CT and MRI, CEUS is radiation-free, minimally invasive, and more cost-effective. However, the technique remains in developmental stages and presents several limitations, including high operator dependency and challenges in whole-liver assessment 9.

To date, no unified consensus exists on the optimal method for accurate and safe liver evaluation. Nevertheless, CEUS has been validated as an effective non-invasive imaging modality for diagnosing and treating liver-related diseases. This review provides an overview of common liver-related diseases and their diagnostic approaches, evaluates the advantages and limitations of CEUS, and discusses its current applications and recent advancements. Additionally, the review explores pressing challenges and future prospects in CEUS-assisted liver evaluation and proposes potential improvement strategies. The ultimate goal is to enhance the accuracy and scope of non-invasive liver assessments, offering a comprehensive reference for the expanded and effective application of CEUS in liver disease diagnosis and treatment.

## 2. Types of Liver-Related Diseases

Liver-related diseases are responsible for approximately 2 million deaths annually worldwide. Of these, 1 million deaths result from complications associated with liver cirrhosis, while the remaining 1 million are attributable to viral hepatitis and HCC 10, 11. Liver diseases are broadly categorized into malignant liver tumors and benign conditions. Benign liver-related diseases encompass a variety of chronic liver conditions, including hepatitis virus infection, alcoholic liver disease (ALD), nonalcoholic fatty liver disease (NAFLD), and cirrhosis, as well as benign liver tumors such as hemangioma, adenoma, and focal nodular hyperplasia (FNH). Malignant liver tumors primarily include HCC, cholangiocarcinoma, hepatoblastoma, and metastatic liver tumors. These diseases pose significant threats to human health globally and may lead to severe consequences if left untreated. Chronic hepatitis often progresses to end-stage liver disease (ESLD), including cirrhosis, HCC, and chronic liver failure, all of which are associated with poor clinical outcomes 12. The following sections briefly introduce common liver-related diseases.

### 2.1. HCC and Liver Metastatic Tumors

This section provides an overview of the epidemiology of HCC and metastatic tumors. HCC is one of the most prevalent and lethal cancers globally, ranking as the second leading cause of cancer-related mortality and the most common primary malignancy of the liver 4, 13. Unlike other common cancers, such as breast and lung cancers, the mortality rate of HCC continues to rise, increasing by 2-3% annually. The poor prognosis and high mortality associated with HCC are frequently attributed to the lack of accurate early diagnostic tools, inadequate monitoring, and the absence of effective treatments for advanced-stage primary liver cancer. Current guidelines recommend the use of ultrasound imaging in combination with serum alpha-fetoprotein (AFP) assessments for early detection of HCC; however, these methods often exhibit insufficient specificity and sensitivity for detecting early-stage disease 14. Therefore, identifying more effective and precise approaches for early HCC detection is imperative. Additionally, metastatic liver tumors are common due to the liver's favorable environment for cancer cells disseminated via the bloodstream. In clinical practice, ultrasound and CT are commonly used to screen for liver metastases, while biopsy pathology remains the standard for confirmation.

### 2.2. Hepatic Hemangioma

Hepatic hemangioma is the second most prevalent benign solid liver tumor and predominantly affects women. It is most commonly detected in individuals aged 30 to 50, although it can occur across a broad age range, including in children 15. Hepatic hemangiomas are often discovered incidentally during B-ultrasound examinations or abdominal surgeries, as they typically remain asymptomatic. Larger hemangiomas may exhibit heterogeneity due to calcification, cystic changes, and fibrotic regions. Consequently, advanced imaging modalities such as Doppler ultrasound are essential for distinguishing hemangiomas from primary benign or malignant liver tumors, as well as metastatic lesions 16.

### 2.3. FNH

FNH is a benign liver tumor and the second most common type of primary liver tumor following hemangioma, accounting for approximately 8% of all primary liver tumors 17. It is highly vascularized and generally asymptomatic, making imaging techniques crucial for diagnosis 18. A characteristic imaging feature of FNH is the "spoke-wheel" sign, which refers to the central artery radiating outward toward the periphery of the lesion 19.

### 2.4. Liver Cirrhosis

Cirrhosis ranks as the 11th most common cause of death globally and, in conjunction with liver cancer, accounts for 3.5% of all deaths. Additionally, it is among the top 20 causes of disability-adjusted life years (DALYs) and life-year losses, contributing 1.6% and 2.1%, respectively, to the global disease burden 10, 11. Liver cirrhosis typically results from factors such as obesity, excessive alcohol consumption, and hepatitis infection. It progresses from an asymptomatic stage to symptomatic stages characterized by progressive portal hypertension, systemic inflammation, and liver failure. Diagnosis of liver cirrhosis commonly involves a combination of imaging techniques (e.g., ultrasonography, CT, or MRI) and serological tests (e.g., transaminase and cholestasis indices), although liver biopsy remains the gold standard for identifying the underlying causes of cirrhosis 20, 21. Given the invasive nature of biopsies, developing safer and more accurate diagnostic alternatives is crucial.

### 2.5. NAFLD

NAFLD encompasses a spectrum of conditions, ranging from mild steatosis to nonalcoholic steatohepatitis (NASH), which may also involve fibrosis and progress to cirrhosis. NAFLD is a significant global public health issue, affecting approximately 25% of the global population 22. In the United States alone, NAFLD incurs an annual economic burden of $103 billion in indirect costs, alongside an additional $188 billion in societal costs 22, 23. These figures underscore the urgent need for cost-effective strategies to monitor disease progression, ensure timely intervention, and alleviate societal and economic burdens.

## 3. Methods for Monitoring Liver-Related Diseases

### 3.1. Medical History and Clinical Manifestation

Liver-related diseases are caused by diverse factors, including viral, bacterial, and parasitic infections, as well as unhealthy dietary habits, excessive alcohol consumption, and other comorbid conditions. For instance, viral infections such as hepatitis B often result in viral hepatitis, while prolonged and heavy alcohol consumption may lead to alcoholic hepatitis. Additionally, a high-fat diet increases the risk of developing fatty liver disease. Symptoms of liver diseases include upper abdominal discomfort, fever, fatigue, nausea, anorexia, and abdominal distension. Clinical signs such as liver tenderness, palmar erythema, spider angiomas, jaundice, and ascites may also be present. While medical history inquiries, symptom evaluations, and basic physical examinations provide preliminary diagnostic insights, they are typically insufficient for identifying specific liver diseases.

### 3.2. Laboratory Examination

Laboratory examinations are pivotal in monitoring the metabolism of proteins, lipids, and bilirubin, as well as in evaluating serum enzymes such as transaminases, which provide insights into the extent of liver cell damage, metabolic function, and the liver's synthetic reserve capacity 24-26. In clinical practice, clinicians select appropriate liver function tests based on the patient's specific condition to ensure a comprehensive and accurate evaluation. Nevertheless, laboratory tests alone are insufficient to confirm the diagnosis of liver-related diseases, requiring corroboration with additional evidence.

### 3.3. Imaging Examination

The primary imaging modalities employed in clinical practice include ultrasound, CT, and MRI. Among these, ultrasound is the preferred and initial diagnostic tool for liver-related diseases. Two-dimensional ultrasound provides information on liver size, morphology, edge characteristics, parenchymal echogenicity, and the status of intrahepatic blood vessels and bile ducts. Doppler ultrasound is instrumental in assessing blood flow dynamics within lesions, while CEUS facilitates quantitative analysis of blood flow perfusion in diseased tissues.

CT is another essential imaging modality, with plain CT scans being effective in detecting a broad spectrum of liver conditions, such as liver cysts, fatty liver, and cirrhosis. Enhanced CT is particularly valuable for evaluating liver vasculature when plain scans are inconclusive. MRI, often used as a supplementary imaging technique following ultrasound and CT, is primarily employed for the differential diagnosis of liver diseases.

Compared with CT and MRI, CEUS offers similar capabilities in observing tissue blood flow perfusion but is distinguished by its higher efficiency, convenience, and the use of safer contrast agents.

### 3.4. Pathological Examination

Pathological examination is universally recognized as the gold standard for diagnosing liver lesions. This method evaluates the type and severity of liver steatosis, assesses ischemia-reperfusion injury during liver transplantation, and provides information on donor liver necrosis, inflammation, fibrosis, and bile stasis 24, 27, 28. Despite its diagnostic accuracy, liver biopsy is associated with several limitations, including high cost, the need for specialized expertise, and the invasive nature of the procedure, which can cause discomfort or harm to patients. Additionally, sampling inaccuracies can result in misdiagnoses. Consequently, many patients are reluctant to undergo liver biopsy 29.

## 4. Introduction to CEUS

### 4.1. Principle

Ultrasound imaging utilizes pulses of sound waves to generate visual representations of reflected signals from tissues. CEUS, also known as acoustic contrast, employs specialized contrast agents to enhance backscattered echoes in ultrasound imaging. Ultrasound-specific contrast agents (UCAs) consist of microbubbles with a gas core encapsulated by a stabilizing shell. Upon intravenous injection, these UCAs remain confined within the bloodstream, enhancing the resolution, sensitivity, and specificity of ultrasound imaging.

Currently, four UCAs are approved for liver imaging: Definity/Luminity (Lantheus Medical Imaging, North Billerica, MA, USA), SonoVue/Lumason (Bracco Suisse SA, Geneva, Switzerland), Optison (GE Healthcare AS, Oslo, Norway), and Sonazoid (GE Healthcare AS, Oslo, Norway) 30. The optimal dosage of contrast agents is influenced by factors such as the type of UCA, ultrasound equipment, target lesion, and patient-specific characteristics, including size and age. For most liver indications, SonoVue is administered at a dosage of 2.4 mL with a mechanical index (MI) of 0.06-0.10. Sonazoid is used at 0.015 mL/kg with an MI of 0.18-0.22, while Definity™ and Optison™ are administered at a standard adult dosage of 0.2-0.3 mL 31, 32.

Prior to imaging, preparatory steps include determining the patient's optimal positioning, identifying the target lesion, and selecting the most suitable scanning plane along the axis of respiratory motion (commonly longitudinal) to minimize motion artifacts caused by respiration 31. During CEUS, a timer is initiated, and the contrast agent is slowly injected intravenously over 2-3 seconds, based on patient weight and contrast volume. A saline flush of 5-10 mL at a rate of approximately 2 mL/s is immediately administered to clear the line 31, 33.

The scanning process commences with a focused examination during the initial arterial and early portal venous phases within the first 60 seconds after contrast administration. Subsequent scanning is performed intermittently every 30 seconds for approximately six minutes or until the contrast agent has completely washed out from the surrounding liver tissue 7, 8.

Due to the dual blood supply of the liver (hepatic artery and portal vein), CEUS imaging of the liver exhibits three overlapping vascular phases following UCA administration: the arterial phase, portal venous phase, and late phase. These phases may exhibit slight temporal variations, particularly in patients with conditions such as heart failure or vascular liver disease 30.

The use of UCAs enables the precise assessment of blood flow perfusion within specific regions of interest without interference from extravascular accumulation of contrast in adjacent tissues. Non-enhancing structures and vascularized tissues can be clearly differentiated, as the brightness of the grayscale image depends on acoustic impedance differences among tissues. Structures with significant impedance disparities produce stronger reflections, appearing brighter in the image. Conversely, blood, primarily composed of erythrocytes, generates minimal backscatter and appears dark in grayscale imaging 34. The microbubbles in UCAs enhance blood backscattering, improving imaging clarity 35. This characteristic makes UCAs highly effective in enhancing the diagnostic accuracy of ultrasound imaging, particularly in evaluating blood flow perfusion.

### 4.2. Development

Early research demonstrated that small bubbles in condensed media, such as water or soft tissue, could produce robust ultrasonic echoes. Furthermore, the ultrasonic field itself could generate "cavitation bubbles," offering a novel approach to enhancing image contrast 36. In 1968, Gramiak and Shah first reported that the injection of indocyanine green solution during aortic root examination enhanced contrast due to the presence of small bubbles in the injected liquid 37. Subsequently, in 1984, Feinstein *et al.* discovered that microbubbles produced during ultrasound treatment were smaller, more uniform, and more stable, facilitating the passage of contrast agents through the capillary bed and enabling enhancement of the left heart cavity after intravenous injection 38. The acronym CEUS was introduced by members of the European Federation of Societies for Ultrasound in Medicine and Biology (EFSUMB) 39. Initially developed to enhance Doppler signals, CEUS has evolved to incorporate advanced contrast-specific techniques. Over time, continuous advancements in contrast agents and imaging technologies have contributed to its widespread clinical application.

Currently, CEUS is extensively utilized for assessing blood flow in solid organs such as the heart, liver, kidneys, and breast, enabling precise evaluation of blood perfusion in both healthy and diseased tissues. Cardiac CEUS, introduced in the late 1960s, has undergone rapid development and is now employed in diagnosing congenital heart disease, valve disorders, myocardial perfusion evaluation, and detecting cardiac thrombi and masses 40. Additionally, CEUS holds significant potential as a non-invasive imaging technique for evaluating benign and malignant breast lesions 41. It is also used to assess kidney quality, particularly in kidney transplantation. In pediatrics, CEUS is applied to evaluate focal liver lesions (FLLs), perform echocardiography through intravenous administration, and assess bladder ureteral reflux via intravesical application 42. Extensive literature indicates that liver lesion evaluation constitutes the primary indication for CEUS in both research and clinical practice 43. Consequently, this review focuses on the clinical applications of CEUS in diagnosing and managing liver-related diseases.

### 4.3. Advantages

#### 4.3.1. Safety

When ultrasound fails to detect blood flow, alternative imaging techniques such as computed tomography angiography (CTA), magnetic resonance angiography (MRA), and catheter angiography are typically employed to confirm findings and assess the extent of thrombosis 44. However, these methods require the administration of intravenous contrast agents, which carry the risk of allergic reactions. Both iodinated and gadolinium-based contrast agents are associated with such adverse effects. Furthermore, MRI examinations are time-consuming, making them unsuitable for critically ill patients, while catheter angiography, despite being the gold standard for vascular assessment, involves procedural risks.

In contrast, CEUS is particularly valuable for patients unable to undergo the aforementioned imaging modalities or when their results are inconclusive 34. Compared to traditional contrast agents, UCAs have a low incidence of adverse reactions, including serious allergic responses, rendering pre-examination laboratory tests generally unnecessary 42. A large retrospective study of 34,478 cases reported only 40 adverse reactions (0.12%), including three cases of anaphylactic shock (<0.01%) 45. Additionally, UCAs exhibit no nephrotoxicity or hepatotoxicity, are rapidly eliminated from the body without deposition, and can be administered multiple times during a single examination without an absolute dosage limit 34, 46.

The non-invasive and safe nature of CEUS enhances patient acceptance compared to biopsy, which carries inherent risks and complications. CEUS is increasingly recognized as a non-invasive alternative for evaluating vascular conditions and can replace multiphase CT and MRI in numerous scenarios. This is particularly advantageous for pediatric patients, who are more susceptible to ionizing radiation and may require sedation or general anesthesia for MRI examinations 46.

#### 4.3.2. Convenience

CEUS offers inherent advantages due to its portability and accessibility 46. Unlike other imaging techniques, CEUS does not require sedation, anesthesia, or exposure to ionizing radiation, allowing it to be performed at the bedside or in other convenient locations. Furthermore, CEUS facilitates the immediate characterization of lesions identified during routine ultrasound, reducing the waiting time associated with CT or MRI imaging and expediting patient management 6. CEUS also permits the simultaneous evaluation of multiple lesions, provides repeatability for follow-up assessments, and allows reinjection of contrast agents to enhance imaging quality 47, 48.

#### 4.3.3. Accuracy

CEUS employs pure intravascular contrast agents, offering excellent spatial resolution for distinguishing non-perfused structures from vascular lesions. It is particularly effective in determining whether lesions are benign or malignant and identifying potential metastases. CEUS also provides real-time, continuous imaging with high resolution and a superior signal-to-background ratio, minimizing interference from timing or respiratory movements 34. Its diagnostic accuracy in detecting resectable tumors is comparable to that of CT and MRI, including for small tumors (≤3 cm) 49. Additionally, CEUS is invaluable in characterizing uncertain FLLs identified by CT, MRI, or positron emission tomography (PET) 30, 50.

#### 4.3.4. Cost-Effectiveness

CEUS is a cost-effective alternative to other liver imaging techniques such as CT and MRI. Economic analyses have demonstrated that CEUS is a more affordable option than contrast-enhanced MRI (CEMRI) while maintaining comparable efficacy. Research indicates that CEUS costs approximately £379 less than contrast-enhanced CT (CECT) for monitoring liver cirrhosis and characterizing FLLs 51. Moreover, CEUS facilitates real-time evaluations post-treatment to confirm therapeutic efficacy, reducing the likelihood of unnecessary continued treatments and lowering overall follow-up and retreatment costs.

### 4.4. Disadvantages

#### 4.4.1. Potential Risks

Although UCAs are generally considered safe and devoid of nephrotoxic or hepatotoxic effects, their use may still pose certain potential adverse events. The primary adverse events associated with intravenous administration are allergic reactions, which are correlated with the UCA dosage and the actual number of microbubbles in the injection volume 42. For intravesical use, adverse events are primarily linked to bladder catheterization procedures. Particular caution should be exercised when using UCAs in vulnerable populations, including lactating or pregnant women and children. Table 1 outlines the contraindications for the use of specific UCAs in children 42.

Patients with cardiac shunts (e.g., right-to-left, bi-directional, or transient right-to-left shunts) are contraindicated from using all second-generation UCAs, as the microbubbles could bypass the pulmonary capillary bed and directly enter the arterial system, potentially causing arteriolar ischemic or cerebral neurovascular events 42, 52. Relative contraindications include acute coronary syndrome, unstable ischemic cardiac disease, worsening congestive heart failure, and severe ventricular arrhythmias. It is crucial to screen patients for any prior hypersensitivity reactions to UCAs before the examination 34.

During intravenous UCA administration, it is essential for radiologists and other involved personnel to remain vigilant and prepared to manage serious adverse events, such as anaphylaxis. In the United States, it is recommended that a resuscitation cart be readily accessible in or near facilities where CEUS examinations are conducted to ensure immediate intervention if necessary 42. Although UCAs are associated with a low incidence of adverse reactions, it is imperative to exclude contraindications and implement appropriate protective measures when using them.

#### 4.4.2. Operator Dependency

The administration of UCAs during CEUS necessitates venous access, a relatively novel procedure for many ultrasound practitioners that requires a learning curve for proficiency. Additionally, CEUS examinations often require two operators: one to acquire images and another to administer the UCAs. This requirement may disrupt the workflow in busy ultrasound units 7, 46.

Moreover, the washout time of contrast agents from the background parenchyma varies depending on the ultrasound machine, transducer, and type of contrast used. This duration can range from as early as 4 minutes to as late as 10 minutes 7, 8. The accuracy of CEUS results is closely tied to the operator's expertise and the quality of the equipment used 53. Therefore, training highly skilled operators and developing standardized, high-quality equipment are essential for the widespread adoption of CEUS in clinical practice.

#### 4.4.3. Interference Factors

Similar to conventional ultrasound, CEUS imaging can be influenced by various factors, such as wound dressings, patient mobility limitations, body habitus, lesion size and location, and gas from postoperative pneumoperitoneum or overlying intestines 53, 54. These interferences can diminish the precision of CEUS assessments. Future advancements in technology are expected to mitigate these limitations, thereby improving the applicability and accuracy of CEUS.

In summary, CEUS presents both significant advantages and notable disadvantages. Table 2 provides a detailed comparison of these aspects 9, 34, 55. A comprehensive understanding of the benefits and limitations of different imaging modalities enables healthcare professionals to make informed decisions tailored to each patient's specific needs and circumstances.

## 5. Clinical Practice of CEUS in Liver-Related Diseases

**Figure 2:**
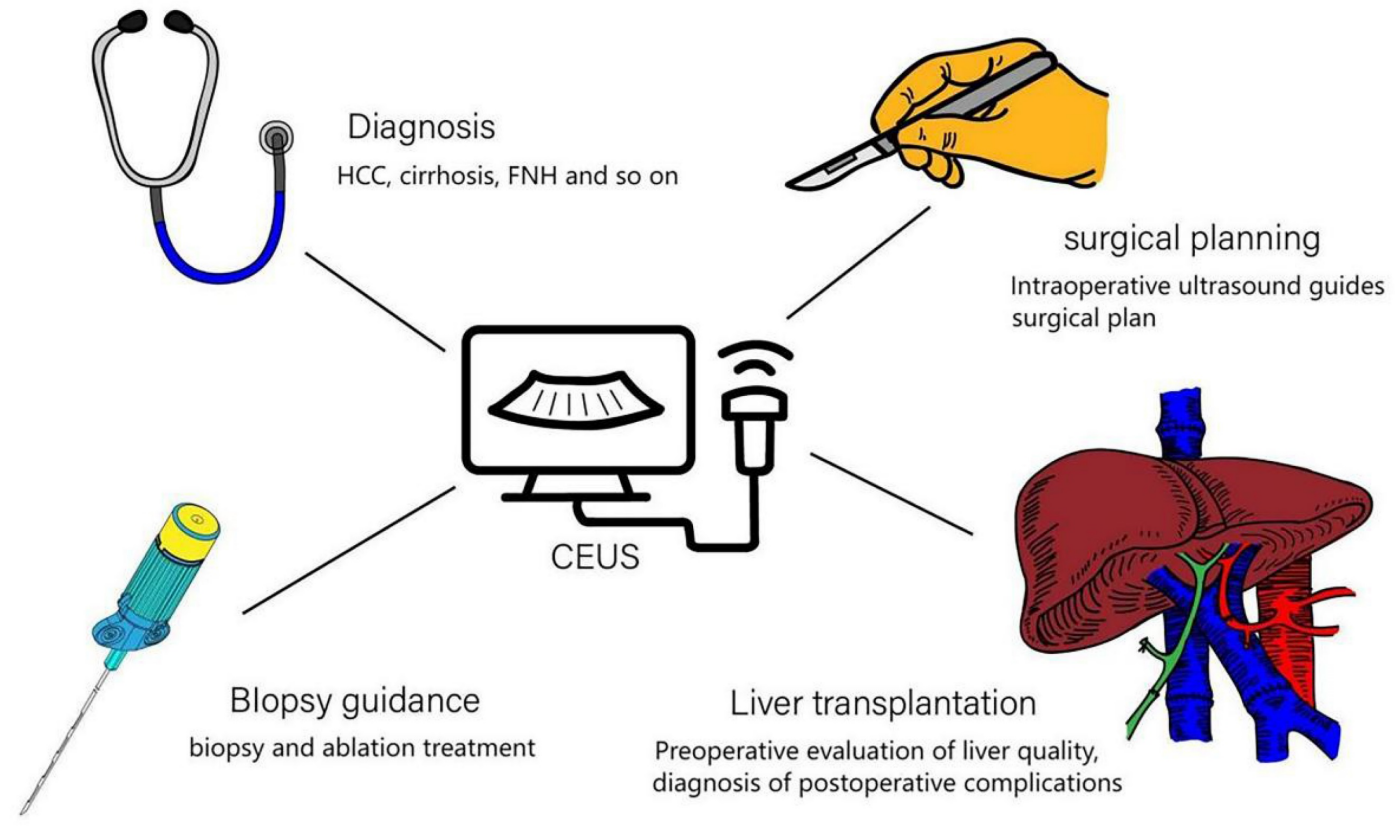
** Clinical Applications of CEUS in Liver Diseases.** CEUS can assist in diagnosing liver-related diseases, guide local treatments such as biopsy, support surgical planning in the operating room, and evaluate the liver during transplantation procedures.

CEUS has extensive applications in clinical practice. It allows for the observation of the shape, internal structure, and spatial relationships of liver lesions, along with morphological evaluation of the liver and portal vein circulation 34. These capabilities make CEUS an invaluable tool for diagnosing liver-related diseases and monitoring treatment outcomes, such as in cases of liver cancer and cirrhosis. Additionally, CEUS facilitates clinical procedures, including needle biopsies and radiofrequency ablation. It also plays a crucial role in liver transplantation by assessing donor liver quality and size, while aiding surgeons in adjusting surgical plans during procedures. Common clinical applications of CEUS in liver diseases are illustrated in Figure 2.

### 5.1. Diagnosis of Liver-Related Diseases

CEUS is particularly effective in diagnosing focal liver disease, especially in cases where CECT or CEMRI results are inconclusive or contraindicated 34. For example, high-risk patients for HCC who cannot undergo other imaging procedures due to safety concerns, such as impaired renal function, can benefit from CEUS for the evaluation of FLLs 51.

**Figure 3:**
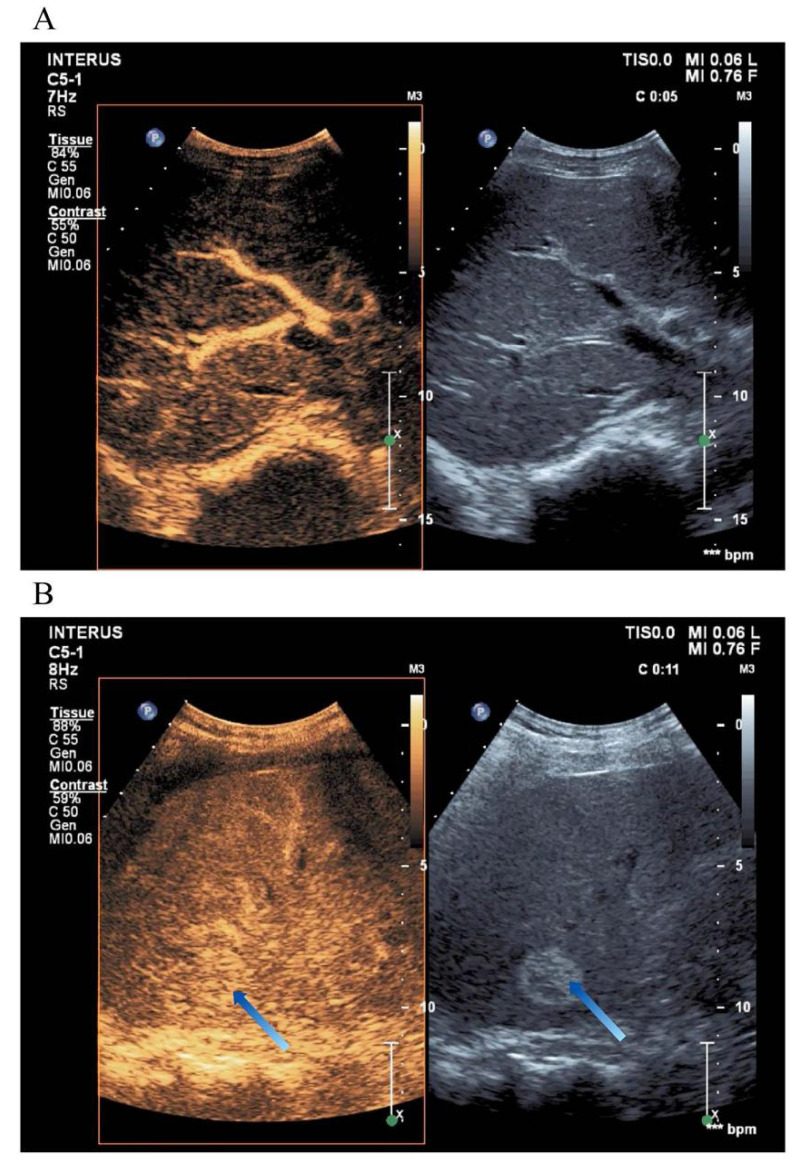
** Liver Contrast-Enhanced Ultrasound Images.** (A) CEUS provides a more detailed visualization of the liver's blood flow trajectory compared to conventional ultrasound imaging. (B) The arrow indicates a distinctly delineated, highly echogenic elliptical area, representing a liver hemangioma.

In the liver, CEUS enables the detailed visualization of blood flow through the portal vein, hepatic artery, hepatic vein, and bile ducts, aiding in the diagnosis of liver parenchymal lesions 35. As a real-time imaging modality, CEUS exhibits higher sensitivity than Doppler ultrasound, dynamic CT, MRI, hepatic angiography, or liver scintigraphy 21. Table 3 summarizes the enhancement patterns observed in common liver diseases using CEUS, and Figure 3 illustrates representative liver evaluation images obtained through CEUS 9, 46.

Studies have shown that the timing and intensity of contrast washout can help distinguish hepatocellular from non-hepatocellular malignancies. For instance, early washout (within 60 seconds after contrast injection) or marked washout (complete contrast disappearance within 2 minutes) is often observed in liver metastases and intrahepatic cholangiocarcinoma. In contrast, HCC typically exhibits late washout (occurring more than 60 seconds after injection) and mild contrast disappearance 9, 46, 56-58. Benign lesions do not exhibit washout and may display distinct arterial phase enhancement patterns, facilitating accurate diagnosis 34, 46.

Currently, CEUS has gained recognition as a valuable tool for qualitatively analyzing HCC, offering real-time guidance during locoregional therapies and improving diagnostic accuracy for detecting residual tumor activity or recurrence. It has been shown to surpass CECT and MRI in identifying patients requiring additional treatment at earlier stages 53. In 2024, the CEUS non-radiotherapy treatment response evaluation algorithm was incorporated into the LI-RADS system, providing standardized guidance for assessing tumor viability following non-radiotherapy locoregional treatments or surgical resection 59. Qin X *et al.* developed a deep learning model based on CEUS to predict microvascular invasion in HCC, aiding in the optimization of treatment strategies and prognostic assessments for high-risk patients 60.

CEUS has proven effective in characterizing liver malignancies, detecting metastatic lesions, and monitoring treatment outcomes following local ablation surgery in oncology patients. It is also utilized for routine surveillance in patients with chronic liver diseases 61-63. The reinjection technique, involving a second administration of contrast agent, may be employed to confirm the metastatic nature of focal regions exhibiting contrast washout by demonstrating arterial phase (AP) enhancement within these areas 30. Based on arterial phase contrast enhancement, metastatic tumors can be categorized as highly vascular or low-vascular. However, all metastatic tumors share a common feature of contrast agent elution in the portal or late phases, resulting in low lesion enhancement 64.

A study by the National Institute for Health and Care Excellence compared CEUS with CECT and CEMRI in detecting liver metastases in patients with known primary cancers. The findings indicated that CEUS alone might be sufficient to exclude liver metastasis in patients with primary malignant tumors 51, 65. Similarly, a meta-analysis involving 2,646 patients (2,981 lesions) compared CEUS with CECT and CEMRI for diagnosing liver-related diseases. It revealed that CEUS had the highest specificity, while CECT demonstrated the greatest sensitivity. However, no statistically significant differences were observed among the three modalities 51, 66. Further large-scale clinical studies are necessary to confirm whether CEUS could serve as a valid alternative to CT, MRI, or other imaging techniques for accurately assessing liver-related diseases, including malignant liver metastases.

In a clinical study involving 101 liver hemangioma lesions, CEUS demonstrated high diagnostic value for hypoechoic liver hemangiomas, characterized by peripheral nodular or circular enhancement. CEUS imaging of hepatic hemangiomas predominantly features centripetal filling and phase transitions described as "fast in, slow out" or "slow in, slow out" 67. Furthermore, CEUS is particularly effective in detecting the spoke-wheel sign or central scar in FNH, often during the early arterial phase 68. It is also widely employed in diagnosing liver cirrhosis, as it allows real-time identification of the enhanced features of FLLs in cirrhotic patients 21.

Despite its utility, many FLLs remain indeterminate after ultrasound imaging due to the similar appearances of benign and malignant lesions 7. Lesions situated near the diaphragmatic dome or deep within the right hepatic lobe pose significant visualization challenges on CEUS, particularly in large patients or those with hepatic steatosis. These difficulties are consistent with limitations encountered in grayscale ultrasound imaging of the liver. Consequently, CECT and/or CEMRI should be considered for patients with multiple, distinct liver abnormalities, including those with Fontan-associated liver disease, who are at high risk for both benign and malignant lesions. CEMRI is preferred for its ability to comprehensively evaluate both the liver and associated lesions.

Additionally, CEUS alone is insufficient for tumor staging. When malignant tumors are suspected or confirmed via ultrasound, CECT or CEMRI is required to achieve complete imaging-based staging and evaluate the extent of associated diseases 46, 69. Moreover, the short duration of arterial phase enhancement and the limited ultrasound field of view restrict CEUS to the evaluation of only one or two lesions during the arterial phase 7. Comprehensive liver assessment remains challenging, potentially leading to the omission of new lesions distant from the target area 9.

### 5.2 Guiding Local Treatment and Evaluation

In clinical practice, CEUS serves as a valuable imaging modality to enhance the accuracy of lesion localization and evaluate therapeutic outcomes following medical interventions. A survey indicated that CEUS improves lesion conspicuity and assists in procedural guidance, achieving a technical success rate of 95.2% in biopsy procedures and 69.7% in radiofrequency ablation 70.

CEUS provides significant advantages in guiding percutaneous lesion biopsies by enabling precise targeting of the needle. This minimizes the risk of sampling avascular or hypovascular areas, thereby ensuring accurate needle placement within tumor-involved regions of irregularly shaped organs. It also facilitates the biopsy of extremely small nodules 71. Employing CEUS to guide puncture biopsies reduces the false-negative rate while minimizing harm to the patient caused by the procedure.

Local ablative treatments, including radiofrequency ablation (RFA) and microwave ablation (MWA), have been demonstrated to effectively control tumor growth in patients with low-burden HCC and metastases 72, 73. CEUS is employed to ensure precise tumor localization and enhance real-time image guidance during thermal liver ablation by visualizing tumor perfusion dynamics and detecting residual tumor tissue following microwave ablation 53. Additionally, CEUS enables immediate evaluation of RFA treatment efficacy within the interventional suite. A study by Meloni *et al.* found that CEUS performed 5-10 minutes post-surgery yielded comparable results to CEUS or CECT conducted 24 hours later in the treatment of 94 liver tumors using RFA or MWA 74.

Immediate post-ablation CEUS facilitates validation of treatment success by confirming the absence of nodular enhancement in multi-vessel lesions or the presence of low-perfusion ablation volumes surrounding and exceeding the target lesion in fewer vessel lesions. It can also identify residual disease, enabling re-treatment during the same procedure, thus avoiding delays and reducing the likelihood of incomplete ablation 9. This approach also leads to significant cost savings. In a study involving 93 patients with 148 HCC lesions treated with percutaneous RFA, intraprocedural CEUS for immediate assessment reduced the need for follow-up ablative sessions, resulting in cost savings for both patients and healthcare institutions 75. Consequently, immediate CEUS evaluation following surgery enhances treatment thoroughness and provides economic benefits.

However, post-ablation reactive hyperemia, occurring immediately or within the first 30 minutes after treatment, may obscure viable enhancing tumors, potentially leading to false impressions of tumor persistence due to the irregular contours of the hyperemia 76. Additionally, gas bubbles generated during ablation can obstruct visualization, partially obscuring tumor sections and limiting CEUS sensitivity for detecting incomplete ablation immediately post-treatment 77. Therefore, CEUS conducted during this early post-treatment period is generally not recommended 78. To determine the optimal timing for CEUS evaluation after RFA, further clinical studies with larger sample sizes are necessary to balance evaluation accuracy and economic efficiency.

The efficacy of CEUS in guiding RFA has been well established. Liu *et al.* demonstrated the efficiency and feasibility of using CEUS to guide HCC patients undergoing RFA, achieving an effectiveness rate of 99% 79. Moreover, MDCT scans performed one day after CEUS-guided RF ablation revealed complete tumor ablation in 96% of cases 51. Accurate evaluation of residual tumor tissue and microvasculature is critical for successful local ablation therapy. This requires CEUS imaging during the early vascular phase to ensure negativity of the lesion margin, reducing the need for repeat ablation procedures.

Nevertheless, compared to CECT, intraoperative CEUS has limitations in assessing the entire liver, which may result in the oversight of new lesions located far from the target lesion. Multimodal fusion imaging offers an effective solution for lesion localization and guiding ablation procedures. Fusion 3D imaging also facilitates precise determination of safe margins 80, 81. Furthermore, CEUS can be used post-ablation to detect immediate complications such as hepatic infarction and hemorrhage 53, 82, 83. It also serves as a diagnostic tool for identifying postoperative active bleeding 34.

In addition to providing guidance for biopsy and ablation treatments, CEUS holds substantial potential as a valuable tool for surgical planning in the operating room. It can facilitate intraoperative confirmation of resectability, detect new lesions that may affect the surgical approach, assist in lesion localization, evaluate the integrity of the hepatic vascular and biliary tree, and assess the presence of extrahepatic disease 84. With the growing use of CEUS in surgery, studies have demonstrated its superior capabilities in detecting HCC and colorectal metastases compared to intraoperative ultrasound or even CT 85. Several studies have indicated that contrast-enhanced intraoperative ultrasound findings have led to significant alterations in surgical management in a considerable percentage of cases, ranging from 14.7% to 76% 86-90.

### 5.3. Liver Transplantation

When US, CT, and/or MRI imaging reports show ambiguous vascular and hepatic parenchymal findings, particularly in instances of portal vein overlap or smaller hepatic artery caliber, CEUS can evaluate the anatomical structures of blood vessels in the donor liver, including the hepatic artery, portal vein, and hepatic vein, prior to surgery. This assessment is crucial for liver transplantation, especially for split liver transplantation 91. Vascular complications following liver transplantation have a reported incidence rate of 7% to 30%, leading to increased postoperative morbidity and decreased graft survival, potentially necessitating retransplantation 92. CEUS can detect luminal stenosis or filling defects, facilitating the identification of portal thrombus hypervascularization and distinguishing between HCC portal invasion and bland portal vein thrombosis 30. Furthermore, for transplant surgery patients, CEUS can also be used to visualize fistulas and incomplete anastomoses between adjacent structures. It has been previously reported that injecting contrast agents into the biliary system through a T-tube is more effective than intravenous injection in enhancing the accuracy of CEUS for confirming biliary leaks and strictures 93. Therefore, it is essential to use conventional ultrasound and CEUS for the prompt and accurate detection of biliary and vascular complications following transplantation surgery, thereby increasing the survival rate of transplanted livers.

Additionally, liver parenchymal microcirculation perfusion obstruction can lead to liver tissue ischemia and damage, which negatively impacts the quality of the donor liver. This, in turn, is closely related to the increased incidence of complications following liver transplantation and higher mortality rates in recipients 89, 94. Recent studies have confirmed that low levels of donor liver perfusion, as evaluated by CEUS, increase the risk of early graft dysfunction following liver transplantation 95-97. Consequently, utilizing CEUS to evaluate donor liver parenchymal microcirculation perfusion is essential for assessing donor liver quality. Implementing robust early postoperative ultrasound monitoring allows for the timely detection of issues and facilitates effective intervention treatments, promoting liver function recovery and providing significant clinical value.

Moreover, liver transplantation surgery has stringent requirements for the quality of the donor liver, particularly in split liver transplantation, where only non-fibrotic donor livers are suitable. The degree of fatty degeneration in the donor liver is also an independent risk factor influencing the early prognosis of split liver transplantation 26. According to relevant literature, ultrasound has demonstrated a sensitivity of 0.812-0.849 and a specificity of 0.600-1.000 in diagnosing moderate and severe steatosis 98-101. CEUS can be used to non-invasively evaluate the extent of liver fibrosis in the donor liver 102. Thus, CEUS can be applied to assess liver steatosis and fibrosis levels before transplantation, aiding in the selection of more suitable liver donors and ensuring the long-term effectiveness of the donor liver in the recipient.

Finally, CEUS can also be utilized to assess the volume and quality of the donor liver. Relevant literature indicates that in both split liver transplantation and live donor liver transplantation, donor liver volume is smaller for increased donor safety; however, a larger donor liver volume is more beneficial for the recipient, as it greatly reduces the risk of small liver syndrome 103. This conflicting supply-demand situation underscores the importance of accurately assessing donor liver volume. The literature suggests that the volume of the left and right portions of the donor liver can be evaluated through the diameter and blood flow parameters of the left and right portal veins, which are useful in determining the residual liver volume in living or split liver transplantation. Additionally, preoperative evaluation of donor liver volume can be accomplished using three-dimensional ultrasound reconstruction technology 104, 105.

### 5.4. Evaluating Trauma

In cases of trauma or postoperative complications, CEUS can accurately identify the location and nature of hemorrhagic lesions, thereby facilitating timely interventions. Research has indicated that CEUS is a promising modality for assessing active abdominal bleeding 106, 107. Given that UCAs remain intravascular, the presence of microbubbles in the peritoneal or retroperitoneal space, regions external to organs or vessels, signifies active extravasation and primarily indicates bleeding 108. Although its utility in evaluating active bleeding is somewhat limited, CEUS shows greater potential than CECT in detecting and staging blunt liver injuries. This suggests that CEUS may be particularly beneficial for stable patients who have experienced low-energy trauma 109, 110.

### 5.5. Assisting Other Treatments for HCC

A study reported the development of a nano-ultrasound contrast agent (arsenic trioxide (ATO)/PFH NPs@Au-cRGD) that integrates diagnostic and therapeutic functions. This agent achieves more efficient ultrasound imaging and liver cancer treatment through ferroptosis and chemo-photothermal therapy compared to conventional drugs. When combined with PD-L1 antibodies, the ATO/PFH NPs@Au-cRGD nano-drug delivery system effectively inhibits in situ liver tumors and activates systemic immune responses to suppress lung metastases 111.

Furthermore, research has demonstrated that specific CEUS parameters, such as rise time, peak intensity, and area under the curve, are valuable in evaluating lymph node metastasis, pathological staging, and treatment responses in HCC 112. CEUS-guided transarterial chemoembolization (TACE) for HCC has been shown to effectively inhibit tumor angiogenesis, control tumor progression, extend patient survival, and improve overall prognosis 113.

## 6. Conclusion and Outlook

This review summarizes liver-related diseases, their diagnostic methods, and the clinical applications of CEUS in this domain. As highlighted, CEUS is a non-invasive imaging modality for liver lesion evaluation. The intravascular nature of UCAs provides a unique means of assessing the vascular distribution of FLLs. CEUS offers real-time imaging with high resolution and a superior signal-to-background ratio, making it invaluable for same-day troubleshooting in both inpatient and outpatient settings 34.

In addition to diagnosing liver lesions, CEUS aids in guiding clinical interventions, including biopsy guidance and ablation procedure monitoring, with immediate evaluation of treatment efficacy during interventions 51. It also serves as a tool for assessing donor liver quality before transplantation, monitoring post-transplantation outcomes, assisting surgical planning, and evaluating trauma cases. Como *et al.* proposed a comprehensive diagnostic workflow for liver transplant patients utilizing CEUS. However, the limited availability of relevant literature restricts the ability to quantify the additional value of CEUS when used alone or in combination with other imaging modalities. Consequently, the application of CEUS in liver transplantation remains relatively constrained 114.

Given its low cost, safety, portability, and high temporal resolution, CEUS is an ideal imaging modality for monitoring HCC responses to locoregional therapy 53. It serves as a low-risk adjunct to ultrasound for diagnostic purposes, offering immediate information to support comprehensive diagnoses and guide subsequent therapeutic interventions 35, 54. Additionally, CEUS demonstrates technical superiority over CT and MRI in detecting and characterizing small liver lesions due to its superior resolution. Its real-time imaging capability provides distinct advantages over the static, prospectively oriented contrast-agent examinations conducted with CT or MRI 35, 115.

With advancements in technology, numerous novel techniques based on ultrasound contrast imaging have emerged. Dynamic CEUS (DCE-US), utilizing VueBox, serves as an effective tool for providing quantitative and objective parameters of hepatic tumor vascularization and holds significant potential as a diagnostic imaging modality for future tumor treatment monitoring 116. Shen *et al.* developed a non-invasive predictive nomogram model that integrates image features from Sonazoid CEUS and shear wave elastography with clinical characteristics, thereby significantly enhancing diagnostic performance for FLLs 117. As an innovative imaging method incorporating artificial intelligence, 3D-CEUS demonstrates high sensitivity in assessing postoperative recurrence in liver cancer patients undergoing RFA 118. Additionally, a novel computer-aided color parametric imaging technique for CEUS non-invasively provides valuable hemodynamic information regarding FLLs and predicts the prognosis of HCC patients undergoing RFA treatment 119.

However, several limitations of CEUS require improvement. The primary constraints of CEUS are similar to those of conventional ultrasound, including operator dependence, reduced penetration in patients with a large body habitus or fatty liver, limited visualization of deep or small lesions, and the need for patient cooperation, particularly the ability to hold their breath during the examination 120. Furthermore, adverse events, such as contrast agent allergies, may occur, and CEUS imposes higher demands on operators and equipment compared to traditional ultrasound. A unique limitation of CEUS is its time sensitivity during the arterial phase examination, which confines the evaluation to a single target using a single injection 9. Current CEUS guidelines established by the World Federation for Ultrasound in Medicine and Biology (WFUMB) do not recommend its routine use for monitoring patients at risk of HCC or for staging HCC 121. Another study also highlighted the preference for CT over CEUS in post-RFA ablation monitoring, despite the apparent advantages of CEUS in detecting tumors and assessing post-ablation outcomes 35.

To enable CEUS to replace CT as the preferred method for diagnosing liver diseases such as HCC or monitoring post-local treatments like RFA, strategies must be developed to address its current limitations and to substantiate its clinical value over other conventional imaging methods.

At present, ultrasound plays a well-established role in the diagnosis and treatment of liver-related diseases. It is anticipated that CEUS will gain wider adoption for liver assessment in the future. To achieve this, several challenges need to be addressed. First, the operator dependency of CEUS limits diagnostic accuracy, as it relies heavily on the operator's proficiency and the quality of the equipment. To ensure accurate CEUS evaluations, professional imaging operators should undergo standardized training and assessment, and equipment must be routinely inspected and maintained.

Another limitation is the inability of CEUS to simultaneously evaluate multiple liver lesions within a single field of view. A study suggests two approaches to overcome this limitation: additional UCA injections for evaluating other lesions after the initial injection, or transducer sweeps through the liver in transverse and sagittal planes during the dynamic phase to identify washout—a key malignancy indicator 122. Nevertheless, it remains crucial to address technical challenges to expand the observation range of CEUS or even achieve whole-liver assessment with a single injection.

Additionally, the current application of CEUS in diagnosing and treating liver-related diseases is not yet fully mature. Controversies persist regarding its potential to replace CT or MRI as a routine detection method, as well as its optimal monitoring time post-RFA. Beyond technological advancements, sufficient research evidence is essential to resolve these disputes. For instance, large-scale clinical studies are necessary to explore the consistency of CEUS indicators with liver pathological biopsy findings and their prognostic correlations. Based on such evidence, guidelines should be developed to standardize the use of CEUS in liver assessment.

In summary, conventional liver evaluation methods have inherent limitations, necessitating the adoption of innovative techniques for comprehensive and safe assessments. CEUS not only holds diagnostic significance in traditional liver diseases but also demonstrates immense clinical potential in areas such as liver transplantation, surgical guidance, and postoperative monitoring. The growing body of research on CEUS applications in liver-related diseases underscores the technology's potential. Although further exploration is required, current studies provide valuable reference points for clinical diagnosis and treatment strategies. Future research should focus on large-scale animal and clinical trials to validate existing findings, thus promoting the implementation of practical application protocols. Addressing current technical limitations and gathering robust clinical evidence will enhance the status of CEUS in diagnosing and treating liver-related diseases, ultimately facilitating more effective patient care.

## 7. Limitations

This review has several limitations. Firstly, time constraints precluded the inclusion of recent relevant studies, potentially affecting the timeliness of the conclusions. Secondly, due to length restrictions, the discussion focuses solely on common liver diseases, thereby limiting comprehensiveness. Finally, the conclusions may reflect the author's subjective viewpoints, and bias in interpretation is unavoidable.

## Figures and Tables

**Table 1:** Contraindications of the Three Ultrasound Contrast Agents (UCAs) Currently Used in Children

UCA	Contraindication
SonoVue/Lumason	Hypersensitivity to sulfur hexafluoride lipid microspheres or its components, such as polyethylene glycol (PEG)
Optison	Known or suspected hypersensitivity to perflutren, blood, blood products or albumin
Definity	Hypersensitivity to perflutren lipid microsphere or its components, such as polyethylene glycol (PEG)

Abbreviations: UCA, ultrasound contrast agents.

**Table 2:** Benefits and Drawbacks of Contrast-Enhanced Ultrasound (CEUS)

**Characteristics**	
**Benefits**	**SAFETY** Very few contraindications; Very low contrast allergy rate; High patient tolerance (no need for sedation or general anesthesia); Rapid contrast clearance (can reinject multiple times if necessary. No absolute dose limit); No nephrotoxicity or hepatotoxicity; No ionizing radiation; Non-invasive. **CONVENIENCE** Portable. Can be performed in emergency department, operation room, and intensive care units; No need for sedation or anesthesia; No laboratory screening needed (aside from pregnancy test, when applicable); Imaging possible during patient's free breathing; Same-day evaluation of findings identified during screening/surveillance ultrasound. Can accelerating patient workup. **ACCURACY** Pure intravascular reagents. Can evaluate the vascular distribution of focal liver lesions and determine whether focal liver lesions are benign or malignant and whether there is metastasis; High spatial resolution. Can guide percutaneous biopsy or RF ablation; High signal-to-background using contrast-specific imaging mode; High temporal resolution. Inherent real-time viewing and continuous imaging essentially eliminates risk of contrast mistiming. **LOW COST** A cost-effective replacement for CT and MRI; Allow to immediate assessment of the result of ablation and decrease the number of second ablative sessions.
**Drawbacks**	**ADVERSE EVENTS** Allergic reactions during intravenous use; Adverse events in the process of bladder catheterization during intravesical use. **CONTRAINDICATIONS** Should screen for allergies, recognize and treat reactions if they occur and prepare emergency medications before examination; Use with caution for lactating or pregnant women and children; Absolute contraindications: patient with cardiac shunts(right-to-left, bi-directional, transient right-to-left) ; Relative contraindications: acute coronary syndrome or clinically unstable ischemic cardiac disease, worsening or unstable congestive heart failure, or serious ventricular arrhythmias. **AVAILABILITY** Not yet universally available; Contrast-specific software and skilled sonographer needed; Require two operators perform simultaneously; **APPLICATION LIMITATIONS** Limited assessment of lesion range (only one or two lesions can be evaluated at a single CEUS examination); Not usually appropriate for complete disease staging; Not as accurate as CT and MRI in diagnosing benign and malignant liver lesions. **VISUAL INTERFERENCE** Wound dressings, patient mobility limitations, body habitus and body size; Lesion size and location within the liver, obesity, fatty liver; Some areas of liver may be obscured by ribs, lung, gas generated by postoperative pneumoperitoneum or overlying intestines or bowel gas; Post-ablative reactive hyperemia and gas bubbles produced during the ablation may mask viable enhancing tumor; The results has so much to do with the operator level. **ACCEPTANCE** CEUS LI-RADS not universally embraced by all clinicians, societies, and guidelines at this time; Not yet recognized by OPTN for transplant evaluation; Not yet recommended by LI-RADS for treatment response evaluation.

Abbreviations: LI-RADS, liver imaging, reporting and data system; OPTN, organ procurement and transplantation network.

**Table 3:** Enhancement Patterns of Various Liver Lesions on Contrast-Enhanced Ultrasound (CEUS)

Focal Liver Lesion	Unenhanced	Contrast-Enhanced		
		Arterial Phase 10-45 s	Portal Venous Phase 30-120 s	Late Phase 120 s-6 min
**HCC**	Variable, small lesions hypoechoic, larger lesions heterogeneous	Nonrim hyperenhancement; hypervascularity, disorganized vessels	Sustained enhancement	Mild late washout
**Hemangioma**	Well circumscribed, homogeneously echogenic; atypical hypoechoic	Peripheral discontinuous nodular enhancement, progressive centripetal filling	Centripetal complete or partial fill-in	No washout of enhancing areas
**Adenoma**	Variable; possible presence of hyperechoic fatty or calcific components	Arterially hypervascular	Isoechoic to hyperechoic	Possible washout in late phase
**Mesenchymal** **hamartoma**	Anechoic cyst, isolated or multiple masses	Nonenhancement of cystic components; gradual enhancement of septa and solid components	No washout of enhancing areas	No washout of enhancing areas
**FNH**	Subtle and near isoechoic to liver; central feeding vessel possibly visible	Spoke-wheel, stellate centripetal enhancement, possible enhancing central vessel	No washout; hyperechoic to isoechoic	No washout; hyperechoic to isoechoic
**Hepatoblastoma**	Solitary mass, enhanced echogenicity, disappearance of normal liver morphology	Variable	Mild to marked washout	Marked washout
**Cholangiocarcinoma**	Variable; biliary dilatation	Rim hyperenhancement	Early and/or marked washout	Marked washout
**Metastasis**	Variable	Variably hyperenhancing, rim enhancing	Early and/or marked washout	Marked early washout

HCC: Hepatocellular carcinoma, FNH: Focal nodular hyperplasia.

## Abbreviations

AFP: alpha-fetoprotein
ALD: alcoholic liver disease
CECT: contrast-enhanced CT
CEMRI: contrast-enhanced MRI
CEUS: contrast-enhanced ultrasound
CT: computer tomography
CTA: computed tomography angiography
ESLD: end-stage liver disease
FNH: focal nodular hyperplasia
FLLs: focal liver lesions
HCC: hepatocellular carcinoma
MI: mechanical index
MRI: magnetic resonance imaging
MRA: magnetic resonance angiography
MWA: microwave ablation
NAFLD: nonalcoholic fatty liver disease
NASH: non-alcoholic steatohepatitis
RFA: radiofrequency ablation
UCAs: ultrasound-specific contrast agents
